# Roadblocks to independence: exploring the roles of self-determination and anxiety on daily living skills in autistic transition-aged youth

**DOI:** 10.3389/fpsyt.2026.1774401

**Published:** 2026-03-17

**Authors:** Paige Hemming, Alexandra Kalinyak, Chuong Bui, Susan White

**Affiliations:** 1 Center for Youth Development and Intervention, Department of Psychology, The University of Alabama, Tuscaloosa, AL, United States; 2 Alabama Life Research Institute, The University of Alabama, Tuscaloosa, AL, United States

**Keywords:** adaptive behavior, adulthood, anxiety, autism, daily living skills, self-determination, transition

## Abstract

**Introduction:**

Autistic individuals tend to have lower daily living skills than age-matched peers, and lower skills than what would be predicted by cognitive ability. What is less known are the mechanisms contributing to this profile.

**Methods:**

This project aimed to examine the influence of anxiety and self-determination on low daily living skills (financial management, self-care, and home care) among autistic youth. A partial least squares structural equation model (PLS-SEM) was developed and tested with a sample of autistic transition-aged adolescents and adults (n = 79, ages 16 – 27, M age = 19.41).

**Results:**

Autistic traits were found to have a significant, positive direct effect on anxiety. The only significant direct paths to daily living skills were from self-determination and cognitive ability, with self-determination having positive direct effects on all three daily living skills and cognitive ability positively relating to financial management. The proposed indirect paths were not significant.

**Discussion:**

Self-determination may be a key mechanistic variable for promoting daily living skills in autistic youth, which could have implications for transition-focused supports.

## Introduction

When measuring autistic adult outcomes, adaptive behavior is a key construct, referring to life skills instrumental to quality of life and independent functioning (1). Within this broader category, daily living skills are meant to reflect aspects of self-care, home-care, and community navigation (2, 3). Development of these skills predicts optimal outcomes, such as independent living and working without support, in young adults with intellectual disabilities (4). Many autistic young adults view daily living skills as key to living independently, though the definition of independence may vary (5). However, decades of work have noted a gap between cognitive ability and performance of daily living skills in autistic transition-aged youth (6–9).

Uncovering mechanisms that help to explain why autistic transition-aged youth experience difficulty with daily living skills might inform intervention and programming designed to promote independence, as well as improve quality of life. Promoting independence is especially important during emerging adulthood, which spans late adolescence through one’s twenties. Emerging adulthood has been identified as a period of elevated risk for developing depression and anxiety, as this is a period of instability (of relationships and career) and identity development (10). On top of these concerns, autistic transition-aged youth often have fewer supports than what is required (11) and are without a formal transition plan in place (12), despite potential underperformance or decline of daily living skills in early adulthood (13–15). Understanding what factors contribute to a profile of lower-than-expected daily living skills, in relation to cognitive ability, in autistic emerging adults, would be a first step to improving outcomes related to independence for the 120,000+ autistic youth transitioning into adulthood each year (16). Knowledge of pathways that explain this depressed profile of daily living skills for autistic individuals is critical for developing supports that target this area.

Anxiety may help to explain the relationship between autistic individuals and daily living skills. Autism and anxiety are highly co-occurring conditions; according to a narrative review, around 20% of autistic people have a current diagnosis of an anxiety disorder (17). In a separate systematic review, approximately 42% of autistic adults have had a diagnosis of an anxiety disorder in their lifetime (18). This is greater than the lifetime prevalence of 33% in the general population (19). The difference in prevalence is also seen in emerging adulthood. In one study, nearly two of every three (64.5%) autistic college students, compared to 9.6% of their non-autistic peers, endorsed co-occurring anxiety (20). Even outside of co-occurring diagnoses, a survey study (*n* = 274) found autistic adults were more likely to experience worry compared to non-autistic adults (21). Anxiety is known to prevent individuals from completing tasks out of fear of a negative outcome, even if there is an expected positive outcome from completing the task (22). Since adaptive behaviors are based on what an individual does as opposed to ability (1), it may be that autistic youth are able to complete these tasks but are more hesitant to out of fear of not completing tasks correctly. Considering the prevalence and impact of anxiety on autistic youth, it was theorized that anxiety could be playing a mediating role in the lower frequency of daily living behaviors that is seen with this group.

Self-determination is another potential explanatory factor for challenges with daily living skills in autistic transition-aged youth. Self-determination theory highlights the importance of autonomy through decision-making and goal-setting behaviors (23). Facets of self-determination include, but are not limited to, decision-making, goal setting and attainment, and self-advocacy (24). In previous work on adults in a majority non-autistic sample, higher levels of autistic traits were related to lower levels of self-determination (25). Interviews have also been completed with autistic adults on their experience with self-determination (26). During the interviews, the researchers found that while the participants found self-determination to be important, there were also many barriers to self-determination, such as difficulties in executive functioning and the impact of ableism in their environment. While there is some literature on the importance of self-determination in personal goal achievement (27), little work has been done on the relationship between self-determination and daily living skills. Recent research, however, has found that self-determination in autistic young adults predicts greater caregiver-rated adaptive behaviors, across domains of social interaction, self-care, and meal preparation (28). Overall, an autistic person’s self-determination may impact their ability to complete activities of daily living, and this relationship should be more thoroughly investigated.

Again, due to the strong co-occurrence between autism and anxiety (17), it is important to also consider the potential relationship between anxiety and self-determination. It may be that anxiety impacts self-determination. This has been suggested by Walden and colleagues, who found a negative relationship between general mental health concerns and self-determination in adolescent girls with disabilities, including autism (29). Including this relationship in the study will help parse apart potential self-determination difficulties due to being autistic versus having symptoms of anxiety.

### The current study

This study investigates factors influencing the relationship between autistic traits and frequency of behaviors in three areas of daily living skills (self-care, home care, and financial management) in autistic transition-aged youth. While there is some evidence suggesting that self-determination and anxiety may impact daily living skills in autistic youth, to our knowledge, a model of the interplay of these constructs and autism has not been explored. Examining such a model may provide insight on whether self-determination and/or anxiety is an intermediate in the relationship between autism and daily living skills.

It was hypothesized that autistic traits would be indirectly related to daily living skills through one’s self-determination and anxiety symptoms. We expected higher autistic traits to be associated with higher anxiety symptoms, which in turn would be related to lower frequency of daily living skills. Similarly, it was expected that higher autistic traits would be associated with lower self-determination, which in turn would be related to lower daily living skills.

## Methods

### Participants

Participants (*n* = 79) were recruited for a randomized controlled trial of transition-based services for autistic youth (BLINDED = R34MH131599: White). The sample was predominantly male (74.68%), white (69.62%), and non-Hispanic/Latino (92.41%; see **Table 1**). All participants were transition-aged youth (ages 16 – 27; *M* age = 19.41) with a prior diagnosis of autism spectrum disorder (ASD), which was confirmed by a score of 134 or greater on self-report on the Comprehensive Autism Trait Inventory (CATI; 30) or by a score of 12 or higher on proxy-report on the Social Communication Questionnaire (SCQ; 31). The SCQ was given if the CATI did not meet the cut-off score, to ensure that the prior clinical diagnosis was supported by either self- or proxy-report data. Descriptive statistics for the CATI, along with the other selected measures, are presented in **Table 2**. Participants were excluded from the study if they endorsed suicidal ideation or active psychosis, could not identify someone to complete proxy assessments, or did not meet the comprehension question requirement testing their understanding of the transition-support study (i.e., three of five questions). Although there was no inclusion criterion related to cognitive or verbal ability, participants needed to have sufficient ability to participate in therapy. Procedures were approved by BLINDED = The University of Alabama Institutional Review Board (IRB # 22-07-5746). Participants and their proxy (typically caregivers) assented/consented to participate. They were compensated for their time completing questionnaires.

**Table 1:** Sample demographics (*N* = 79).

Demographic		Frequency (%)
Gender		
Male		59 (74.68%)
Female		15 (18.99%)
Non-binary		3 (3.80%)
Prefer not to answer/missing data		2 (2.53%)
Race		
White/Caucasian		55 (69.62%)
Black/African American		16 (20.25%)
Asian		1 (1.27%)
More than one race		4 (5.06%)
Prefer not to answer/missing data		3 (3.80%)
Ethnicity		
Non-Hispanic/Latino		73 (92.41%)
Hispanic/Latino		2 (2.53%)
Prefer not to answer/missing data		4 (5.06%)
WASI-II standard score ranges		
Extremely Low (69 or below)		8 (10.13%)
Very Low (70 – 79)		5 (6.33%)
Low Average (80 – 89)		13 (16.46%)
Average (90 – 109)		35 (44.30%)
High Average (110 – 119)		9 (11.39%)
Very High (120 – 129)		6 (7.59%)
Extremely High (130 or above)		3 (3.80%)
REALS home care distribution of T-scores		
≤ 29		3 (3.80%)
30 – 39		12 (15.19%)
40 – 44		22 (27.85%)
45 – 54		28 (35.44%)
55 – 59		6 (7.59%)
60 – 69		8 (10.13%)
≥ 70		0 (0%)
REALS self-care distribution of T-scores		
≤ 29		2 (2.53%)
30 – 39		16 (20.25%)
40 – 44		20 (25.32%)
45 – 54		33 (41.77%)
55 – 59		3 (3.80%)
60 – 69		5 (6.33%)
≥ 70		0 (0%)
REALS finance distribution of T-scores		
≤ 29		14 (17.72%)
30 – 39		22 (27.85%)
40 – 44		25 (31.65%)
45 – 54		13 (16.46%)
55 – 59		1 (1.27%)
60 – 69		4 (5.06%)
≥ 70		0 (0%)
**Demographic**	**Range**	**Mean (SD)**
Age	16 – 27	19.41 (2.82)

**Table 2:** Descriptive data.

Measure	Construct	Score type	Range	Mean (SD)
CATI	Autistic traits	Cumulative raw	76 – 203	142.32 (23.75)
DSM-5 CCSM Anxiety	Anxiety	Cumulative raw	0 – 12	3.87 (3.53)
AIR-SD Capacity	Self-determination	Cumulative raw	18 – 60	41.11 (10.10)
REALS Home Care Frequency	Home care	T-score	21.82 – 64.54	46.62 (10.06)
REALS Self-Care Frequency	Self-care	T-score	12.51 – 68.43	46.38 (8.90)
REALS Finance Frequency	Financial management	T-score	25.21 – 62.33	39.65 (9.42)
WASI-II FSIQ-2	Cognitive ability	Standard score	52 – 144	96.73 (19.75)

### Measures

#### Comprehensive autism trait inventory

Autistic traits were measured through the Comprehensive Autism Trait Inventory (CATI; 30). The CATI is a 42-item self-report questionnaire for adults that comprises six subscales: social interactions, communication, social camouflage, repetitive behaviors, cognitive rigidity, and sensory sensitivity (30). This test produces a total score that can range from 42 – 210, with higher scores reflecting higher reporting of autistic traits. The CATI has demonstrated strong internal consistency for the total score and across subscales (α = .81 –.95; 30).

#### DSM-5 level 1 cross-cutting symptom measure

Anxiety symptoms were assessed using the Diagnostic and Statistical Manual of Mental Disorders Level 1 Cross-Cutting Symptom Measure (DSM-5 CCSM; 32). The DSM-5 CCSM is a 23-item screening tool for common mental health symptoms. The current study focuses on reporting within the anxiety domain, which is comprised of three broad anxiety questions: feelings of nervousness, worry, or fear; panic or consistent worry; avoiding anxiety-provoking situations. This assessment produces a cumulative raw score, with higher scores indicating greater anxiety symptoms. Both youth and adult versions of the DSM-5 CCSM are used for this study based on the age of the participant. The DSM-5 CCSM had appropriate reliability during its development for adult (α = .75) and child (α = .69) self-report (33). The anxiety subscale has shown adequate internal consistency within a nationwide sample of college-aged individuals (α = .84; 34). This tool has also been used in studies with autistic adults (35).

#### American institutes for research self-determination scale

Self-determination was measured through the American Institute for Research Self-Determination Scale, Student Form (AIR-SD; 36). The AIR-SD is a 27-item questionnaire that assesses self-determination capacity and opportunity. The capacity and opportunity scales can be combined to create a total self-determination score. The current study used the capacity index to capture participants’ perceived knowledge, abilities, and perceptions surrounding their goal-directed, autonomous behaviors. This scale encompasses 12 items that assess how one engages (‘Things I Do’) and feels (‘How I Feel’) about meeting their needs. The items combine to create a raw subscale score, with higher scores indicating greater self-determination. The AIR-SD yielded adequate internal consistency during its development (α = .91 –.98; 36). It has been used as a self-report measure of self-determination in studies with autistic youth (37).

#### Relationships, employment, autonomy, and life satisfaction measures

Daily living skills were measured through the Relationships, Employment, Autonomy, and Life Satisfaction Measures (REALS; 38). The REALS is a collection of 16 questionnaires that measure areas of adult functioning, developed for autistic adults and adults with other developmental and intellectual disabilities. The REALS was normed on a large sample of adults with intellectual and developmental disabilities, of whom 90% had ASD. The questionnaires are categorized into three domains: relationships, employment, and autonomy. Our model focused on the autonomy domain, which taps into daily living behaviors related to autonomous living, involving taking care of oneself and home, engaging in leisure activities, independently navigating the community, and financial management. Within Autonomy, we focused on home care, self-care, and finance, each assessed using two parallel questionnaires: one measuring the frequency of behaviors and the other measuring level of perceived support. The current study utilizes only the frequency questionnaires to focus on how often the behaviors occur. The questionnaires vary in length: home care and finance each have 4 items, while self-care includes 10 items. Higher T-scores equate to greater frequency of daily living behaviors. Overall, the measure demonstrated adequate internal consistency, including the frequency scales of the autonomy domain used in this study (α = .81 –.83; 39).

#### Wechsler abbreviated scale of intelligence, second edition

Cognitive ability was measured using the Wechsler Abbreviated Scale of Intelligence, Second Edition (WASI-II; 40). The WASI-II is a brief evaluation of intelligence for children and adults (ages 6 – 90). The two-subtest full-scale intelligence quotient (FSIQ-2) was used to estimate cognitive ability, with higher scores reflecting higher levels of cognitive ability compared to age-matched peers. This test was administered virtually using a paper protocol and presentation of the testing items using Microsoft PowerPoint. The FSIQ-2 has strong psychometric properties and is considered a reliable and valid measure of intelligence, with strong internal consistency for adult (α = .94) test-takers (40).

### Procedures

**Figure 1:**
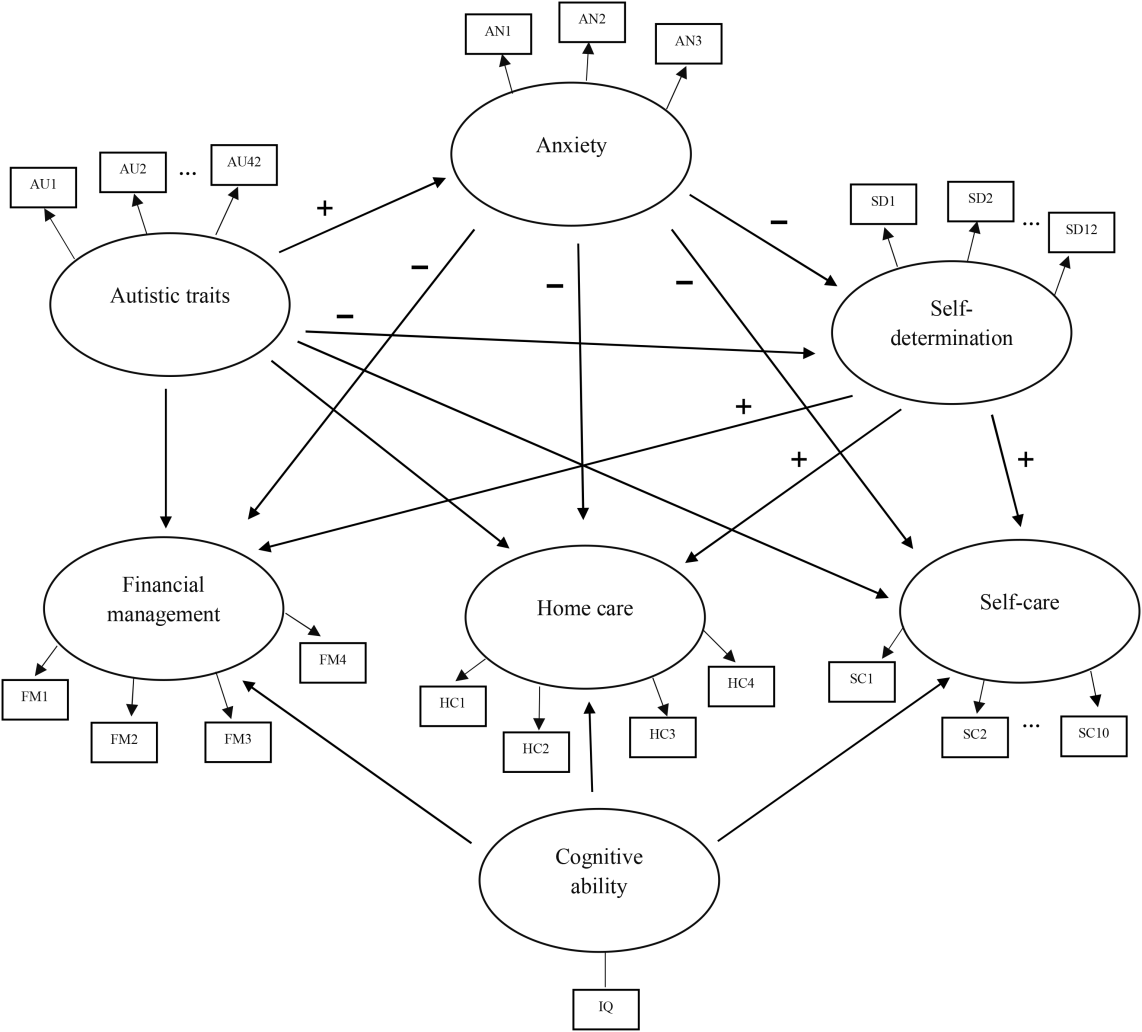
Path diagram depicting hypothesized relationships between constructs.

This cross-sectional study used data collected prior to treatment initiation, collected online through the research electronic data capture platform (REDCap; 41). The hypothesized relationships are summarized in **Figure 1** and were tested using partial least squares structural equation modeling (PLS-SEM). The measurement model contained six multi-item constructs (autistic traits, anxiety symptoms, self-determination capacity, and the three areas of daily living: finance, home care, and self-care), and one single-item construct (cognitive ability). All constructs in this model are considered reflective, meaning experiences with the construct are assumed to be reflected in responses on the indicators (e.g., individuals with higher levels of autistic traits are expected to have higher scores across the related indicators). The structural model specified direct paths from autistic traits to anxiety symptoms and self-determination, and direct paths from anxiety symptoms and self-determination to daily living skills. These paths enabled the testing of the indirect effects of autistic traits on daily living skills through anxiety symptoms and self-determination. In addition, by specifying direct paths from autistic traits to daily living skills, the model controlled for the influence of autistic traits when estimating the direct effects of anxiety and self-determination on daily living skills. To allow for a potential effect of anxiety symptoms on self-determination, a direct path is specified between the constructs. To control for the influence of cognitive ability on daily living skills, direct paths from cognitive ability to daily living skills were also specified.

The model was estimated using the SEMinR package (42) using R Studio, version 2025.09.2 + 418. Bootstrap 95% confidence intervals (5000 bootstrap samples) were used for statistical inference of direct and indirect paths. There were no instances of missing data.

## Results

Participants showed a wide range of cognitive abilities and daily living skills (see **Table 1**). Cognitive ability, measured by FSIQ-2 scores, ranged from Extremely Low to Extremely High. The majority of FSIQ-2 scores (72.15%) fell between Low Average and High Average. The average FSIQ-2 score was 96.73 (SD = 19.75). On the REALS, around 60% of participants had a T-score of 50 or less for home care and self-care (63.29% and 69.62%, respectively) and nearly 90% for financial management (88.61%). A direct comparison of cognitive ability and daily living skills cannot be made due to the standardization differences; the REALS was normed on a developmentally disabled/autistic sample rather than a large, typically developing sample as used for the WASI-II. Given that the majority of the sample (60-90% across domains) obtained T-scores below 50, the participants appear to have daily living skills similar to, or below, what is seen in autistic peers (39).

All measures demonstrated adequate internal reliability (Cronbach’s α >.70, Composite reliability >.70) and discriminant validity (Heterotrait-Monotrait ratios <.85; see **Table 3**). Findings from the indirect paths are displayed in **Table 4**. None of the hypothesized indirect paths between autistic traits and daily living skills attained statistical significance. More specifically, the proposed relationships between autistic traits and the daily living skill areas through anxiety were not supported by the data. Similarly, the proposed indirect paths from autistic traits to the three daily living skills through self-determination were not supported by the data. The indirect paths that involved both anxiety and self-determination (i.e., serial mediators) were also non-significant.

**Table 3:** Construct reliability and discriminant validity.

	Discriminant validity (HTMT ratio)						Reliability	
	Autistic traits	Anxiety	Self-determination	Home care	Self-care	Finance	Alpha	rhoC
Autistic traits	.	.	.	.	.	.	.89	.88
Anxiety	.61	.	.	.	.	.	.84	.90
Self-determination	.37	.17	.	.	.	.	.93	.94
Home care	.27	.09	.43	.	.	.	.84	.89
Self-care	.45	.31	.49	.86	.	.	.78	.82
Finance	.33	.18	.46	.67	.70	.	.74	.83

HTMT, Heterotrait-Monotrait; Alpha, Cronbach’s alpha; rhoC, Composite reliability.

**Table 4:** Bootstrapping results for indirect paths.

Paths	Original est.	Bootstrap Mean	Bootstrap SD	95% CI
Autistic traits → Anxiety → Home care	.050	.048	.098	[-.138;.257]
Autistic traits → Anxiety → Self-care	-.013	-.016	.098	[-.218;.174]
Autistic traits → Anxiety → Finance	.025	.005	.094	[-.196;.187]
Autistic traits **→** Self-determination → Home care	-.170	-.147	.150	[-.390;.225]
Autistic traits **→** Self-determination → Self-care	-.197	-.159	.154	[-.393;.212]
Autistic traits **→** Self-determination → Finance	-.170	-.145	.145	[-.373;.215]
Autistic traits → Anxiety **→** Self-determination → Home care	.038	.027	.046	[-.066;.125]
Autistic traits → Anxiety **→** Self-determination → Self-care	.042	.031	.052	[-.067;.145]
Autistic traits → Anxiety **→** Self-determination → Finance	.037	.027	.048	[-.065;.132]

Original est., estimate from original sample; Bootstrap Mean, average of estimates from bootstrap samples; Bootstrap SD, standard deviation of estimates from bootstrap samples; 95% CI, bootstrap 95% confidence interval.

The direct path results are displayed in **Table 5**. VIF (variance inflation factors) were all below 2.0, which indicated no concerning multicollinearity. The relationship between autistic traits and anxiety was significant (β = .588, 95%CI = [.131,.762], Cohen’s f^2^ = 0.66), with higher reporting of autistic traits relating to higher reporting of anxiety symptoms. All other direct paths from autistic traits were non-significant, including the direct paths to daily living skills.

**Table 5:** Bootstrapping results for direct paths.

Paths	Original est.	Bootstrap Mean	Bootstrap SD	95% CI	f^2^	VIF	R^2^
*Paths → Anxiety*							.38
**Autistic traits**	**.622**	**.588**	**.169**	**[.131;.762]**	**.66**		
*Paths → Self-determination*							.10
Autistic traits	-.416	-.363	.351	[-.745;.590]	.12	1.63	
Anxiety	.144	.109	.175	[-.245;.433]	.01	1.63	
*Paths → Home care*							.16
Autistic traits	-.073	-.089	.260	[-.541;.450]	.00	1.93	
Anxiety	.081	.078	.154	[-.222;.380]	.01	1.76	
**Self-determination**	**.429**	**.396**	**.137**	**[.107;.638]**	**.20**	1.15	
Cognitive ability	.008	.027	.117	[-.210;.247]	.00	1.37	
*Paths → Self-care*							.24
Autistic traits	-.173	-.139	.318	[-.600;.600]	.01	1.93	
Anxiety	-.021	-.027	.156	[-.340;.280]	.00	1.76	
**Self-determination**	**.473**	**.420**	**.152**	**[.064;.658]**	**.27**	1.15	
Cognitive ability	.179	.186	.151	[-.170;.438]	.03	1.37	
*Paths → Finance*							.16
Autistic traits	-.119	-.093	.278	[-.561;.481]	.01	1.93	
Anxiety	.041	.012	.151	[-.307;.283]	.00	1.76	
**Self-determination**	**.410**	**.391**	**.118**	**[.133;.596]**	**.18**	1.15	
**Cognitive ability**	**.236**	**.252**	**.116**	**[.014;.465]**	**.05**	1.37	

Bolded paths indicate significant results. Original est., estimate from original sample; Bootstrap Mean, average of estimates from bootstrap samples; Bootstrap SD, standard deviation of estimates from bootstrap samples; 95% CI, bootstrap 95% confidence interval; f^2^, Cohen’s f^2^; VIF, Variance inflation factor; R^2^, adjusted R^2^.

There were significant direct effects of self-determination on the daily living skills: home care (β = .396, 95%CI = [.107,.638], f^2^ = 0.20), self-care (β = .420, 95%CI = [.064,.658], f^2^ = 0.27), and financial management (β = .391, 95%CI = [.133,.596], f^2^ = 0.18). For the three areas of daily living, higher self-determination was significantly related to greater frequency of the behaviors in that domain.

Anxiety symptoms did not have a significant direct effect on self-determination. The direct paths from anxiety symptoms to daily living skills were also non-significant. Cognitive ability was considered in reference to daily living skills, and it was found to have a significant direct effect on financial management (β = .252, 95%CI = [.014,.465], f^2^ = .05). Cognitive ability did not have significant direct effects on home care or self-care behaviors.

## Discussion

This project sought to elucidate the relationship between autistic traits and daily living skills by testing two indirect pathways through anxiety and self-determination, which are known to have an impact on quality of life for autistic transition-aged youth (25, 43). The current data, however, do not lend support toward the hypothesized roles of anxiety and self-determination. The hypothesized indirect pathways linking autistic traits with the three daily living skills through anxiety symptoms were found to be non-significant. The same is true for self-determination, in that this construct did not reach statistical significance as a mediating factor between autistic traits and adaptive skills. These results suggest that anxiety and self-determination may not be key mechanisms linking autistics traits and daily living skills.

The non-significant indirect paths between autistic traits, anxiety, and daily living skills should be considered within the context of the direct paths. We found that higher self-reported autistic traits were significantly associated with higher levels of anxiety symptoms with a large effect size. There are decades of research on the phenomenon of autism and anxiety (44, 45), though the directional relationship between autism severity and anxiety is less clear. A recent systematic review and meta-analysis examining autistic traits and anxiety symptoms in adults showed mixed findings, with only about half of the studies reporting positive effect sizes (46). An overall significant association between autistic traits and anxiety was not found, with the authors noting high variability across the studies, such that studies with positive effects had relatively larger samples and various autism trait measures were used, most designed for clinical populations, which may limit detection of subclinical autistic traits. Our findings add to this literature and support the association of high autistic traits and anxiety symptoms. We did not see a significant path from anxiety symptoms to self-care, home care, or financial management. Symptoms of anxiety may not impact daily functioning as expected, despite the possibility that anxiety could reduce engagement in daily living behaviors, perhaps due to fear of doing tasks incorrectly (22). Considering the prevalence of anxiety in autistic young adults, it is still important to consider this variable as having a potential impact on the transition to adulthood.

In our study, the indirect paths between autistic traits and daily living skills through self-determination were also found to be non-significant, as was the direct path between autistic traits and self-determination. A different study, however, provided evidence suggesting that higher self-reporting of autistic traits is related to lower levels of self-determination (25). Andrews and colleagues used a majority non-autistic sample (1.9% reported having ASD) to examine whether autistic traits were indirectly related to quality of life through self-determination. Their model supported several indirect paths through self-determination, as well as direct paths from autistic traits to self-determination. Self-determination was measured through a psychological needs satisfaction scale, which evaluates satisfaction with autonomy, competence, and relatedness. Their study assumed that those who reported higher satisfaction would also have higher self-determination. In contrast, the current study, in addition to using a fully autistic sample, taps into an individual’s perceived capacity to complete goal-oriented behaviors, which could be contributing to our difference in findings.

Self-determination was significantly, positively related to frequency of the daily living outcomes (self-care, home care, and financial management), with medium effect sizes. In fact, self-determination was the only construct to significantly relate to each area of daily living skills, other than the link seen between cognitive ability and financial management. This is consistent with recent research indicating that self-determination is related to greater adaptive behaviors (e.g., social interaction, self-care) in autistic young adults (28). This finding may suggest that as one’s ability to engage in goal-directed behaviors and make independent, self-directed decisions improves, engagement with one’s environment is also likely to increase. Cultivating self-determination may be especially important during the transition to adulthood, particularly for autistic youth. Emerging adulthood often presents unique obstacles for autistic youth, with the National Autism Indicators Report revealing that only 58% of autistic young adults had a transition plan put in place by the federally required age and more than 33% did not obtain employment or postsecondary education after high school (12). While the transition to adulthood is challenging for most youth, it may be particularly difficult for those with autism, and fostering self-determination may improve readiness for adult living.

Anxiety symptoms were not related to self-determination. The path was included in the model to examine whether potential difficulties with self-determination were primarily associated with levels of autistic traits or symptoms of anxiety. To our knowledge, the relationship between anxiety and self-determination has yet to be directly explored. In our sample of autistic transition-aged youth, self-determination was not related to anxiety symptoms or autistic traits. Further, the indirect paths from autistic traits to daily living skills through anxiety and self-determination (i.e., serial mediators) were found to be non-significant.

Autistic traits did not significantly relate to any of the three daily living skill areas. Judging by effect sizes, autistic traits appeared to have very small, direct effects on daily living skills. Other recent studies have also found that autistic traits are a weak predictor of daily living skills, and lower levels of these behaviors may be better explained by other factors, such as cognitive ability (6, 8). Perhaps, autism itself may not influence delayed daily living skills; rather, other adjacent factors, such as opportunity or self-determination, may play a larger role. In our model, cognitive ability was significantly related to financial management, with the paths to home care and self-care coming up as non-significant. Higher cognitive abilities may support navigating financial systems and accessing appropriate supports needed for effective financial management. Judging by effect sizes, the effect of cognitive ability on financial management is conventionally considered small, as opposed to the medium effect of self-determination.

There are limitations that should be considered. The data are cross-sectional and observational in nature. Hence, all hypothesized relationships are associations, and no conclusions regarding causation should be made. Some of the measures used are brief (i.e., 3–4 items), which may prevent a comprehensive assessment of the constructs. For instance, the DSM-5 CCSM anxiety scale is used as a screening tool for anxiety disorders and therefore only contains three items. It is possible that a measure that assesses the full range of anxiety symptoms could yield different results. Future work should consider including an anxiety measure that is validated in autistic adult samples. Of similar note, we measured three areas of daily living behaviors that were theorized to be of most interest and relevance to our sample. Other adaptive behaviors (e.g., transportation) may be relevant to the adult transition and daily living that were not included in the model. Further, this project utilized self-report measures, which may introduce bias related to social desirability or memory inaccuracies. A modest sample size could possibly contribute to the absence of evidence of the hypothesized mediated effects. Therefore, the null indirect effects should be interpreted with caution. Lastly, the findings should be interpreted in light of the randomized controlled trial from which the participants were recruited. It is possible that those who sought out the transition-support program present with a particular profile (e.g., lower self-determination) that could differ from a more general sampling of autistic transition-aged youth.

The current study found that one’s perceived capacity to set and work towards goals is linked to how often they engage in daily living behaviors important for autonomy. Frequency of engaging in specific daily living skills was unrelated to autistic traits and anxiety symptoms, making self-determination the sole factor relating to daily living outcomes in this model, apart from cognitive ability contributing to financial management behaviors. Future research must move beyond cross-sectional to longitudinal designs and may explore factors that might mediate the relationship between self-determination and daily living skills, including executive functioning skills and specific mental health conditions. Another next step in research is the development and implementation of programs to boost daily living skills in autistic youth, with self-determination acting as a key mechanism in the intervention.

## Data Availability

The raw data supporting the conclusions of this article will be made available by the authors, without undue reservation.
